# D prostanoid receptor 2 (chemoattractant receptor–homologous molecule expressed on T_H_2 cells) protein expression in asthmatic patients and its effects on bronchial epithelial cells

**DOI:** 10.1016/j.jaci.2014.08.027

**Published:** 2015-02

**Authors:** Sally E. Stinson, Yassine Amrani, Christopher E. Brightling

**Affiliations:** Institute of Lung Health, Department of Infection, Inflammation and Immunity, University of Leicester, Leicester, United Kingdom

**Keywords:** Expression, asthma, immunohistochemistry, prostaglandin D_2_, biopsy, ALI, Air-liquid interface, COPD, Chronic obstructive pulmonary disease, CRTH2, Chemoattractant receptor–homologous molecule expressed on T_H_2 cells, DK-PGD_2_, 13, 14-Dihydro-15-keto prostaglandin D_2_, DP1, D prostanoid receptor 1, DP2, D prostanoid receptor 2, PGD_2_, Prostaglandin D_2_

## Abstract

**Background:**

The D prostanoid receptor 2 (DP2; also known as chemoattractant receptor–homologous molecule expressed on T_H_2 cells) is implicated in the pathogenesis of asthma, but its expression within bronchial biopsy specimens is unknown.

**Objectives:**

We sought to investigate the bronchial submucosal DP2 expression in asthmatic patients and healthy control subjects and to explore its functional role in epithelial cells.

**Methods:**

DP2 protein expression was assessed in bronchial biopsy specimens from asthmatic patients (n = 22) and healthy control subjects (n = 10) by using immunohistochemistry and in primary epithelial cells by using flow cytometry, immunofluorescence, and quantitative RT-PCR. The effects of the selective DP2 agonist 13, 14-dihydro-15-keto prostaglandin D_2_ on epithelial cell migration and differentiation were determined.

**Results:**

Numbers of submucosal DP2^+^ cells were increased in asthmatic patients compared with those in healthy control subjects (mean [SEM]: 78 [5] vs 22 [3]/mm^2^ submucosa, *P* < .001). The bronchial epithelium expressed DP2, but its expression was decreased in asthmatic patients compared with that seen in healthy control subjects (mean [SEM]: 21 [3] vs 72 [11]/10 mm^2^ epithelial area, *P* = .001), with similar differences observed *in vitro* by primary epithelial cells. Squamous metaplasia of the bronchial epithelium was increased in asthmatic patients and related to decreased DP2 expression (*r*_*s*_ = 0.69, *P* < .001). 13, 14-Dihydro-15-keto prostaglandin D_2_ promoted epithelial cell migration and at air-liquid interface cultures increased the number of MUC5AC^+^ and involucrin-positive cells, which were blocked with the DP2-selective antagonist AZD6430.

**Conclusions:**

DP2 is expressed by the bronchial epithelium, and its activation drives epithelial differentiation, suggesting that in addition to its well-characterized role in inflammatory cell migration, DP2 might contribute to airway remodeling in asthmatic patients.

D prostanoid receptor 2 (DP2) or chemoattractant receptor–homologous molecule expressed on T_H_2 cells (CRTH2) is a G protein–coupled receptor that has been implicated in the pathogenesis of allergic diseases.^1^ DP2 is activated by prostaglandin D_2_ (PGD_2_), which is found at high levels in the bronchoalveolar lavage fluid of asthmatic patients.^2-6^ The expression of DP2 and the effects of its stimulation on T_H_2 lymphocytes, eosinophil and basophil migration, and activation has been well characterized.^7,8^ An increase in the number of DP2^+^ inflammatory cells in patients with allergic disease^6^ has highlighted a potential role for this receptor in allergy^9,10^ and asthma.^11,12^ In addition, considerable interest in the development of DP2 antagonists in both patients with allergic conditions^9,10^ and asthmatic patients^11-13^ has strengthened the linkage between the DP2 receptor and inflammatory-related disorders. To date, limited efficacy has been demonstrated for DP2 antagonists in asthmatic patients^11,13^; however, it remains to be determined whether DP2 antagonism is more effective in a subset of patients. A thorough understanding of DP2 expression within the airways and whether changes in receptor expression correlates with disease severity might aid in identifying a responsive asthmatic group. Unfortunately, there is currently a lack of data describing the protein expression of DP2 in bronchial biopsy specimens in asthmatic patients, which has limited the cell types that DP2 function has been explored within. In contrast, the expression of DP2 on epithelial cells has been described from a variety of tissues, including the nose,^14^ skin,^15^ and retina,^16^ and bronchial epithelial cells in patients with chronic obstructive pulmonary disease (COPD).^17^ These studies highlight the potential that DP2 might be expressed on epithelial cells within the airways of asthmatic patients. In addition, in mouse challenge models^18,19^ DP2 antagonists caused a reduction in goblet cell hyperplasia, suggesting that DP2 activation on epithelial cells might play a key role in the pathogenesis of asthma. Comparative studies looking at the expression of DP2 on biopsy specimens from asthmatic patients and healthy control subjects would provide useful data to indicate possible target cells located within the airways for DP2 antagonists, helping to focus future DP2 antagonist study readouts and patient populations.

We hypothesized (1) that submucosal inflammatory cells and the bronchial epithelium express DP2 and that its expression is increased in asthmatic patients and related to disease severity and (2) that activation of DP2 in primary epithelial cells promotes migration and differentiation. To test our hypotheses, we have undertaken an immunohistochemical analysis of bronchial biopsy specimens from asthmatic patients and healthy control subjects and studied the expression and function of DP2 in primary epithelial cells in submerged and air-liquid interface (ALI) cultures.

## Methods

### Subjects

Healthy control subjects and asthmatic patients were recruited from Glenfield Hospital, Leicester, United Kingdom. Asthma severity was defined according to the Global Initiative for Asthma treatment steps.^20^ Subjects were characterized in terms of demographics, smoking history, spirometry, sputum cell counts, and atopic status, which was defined as either 1 or more positive skin prick test responses or blood-specific IgE levels to common aeroallergens. Healthy subjects had no history of respiratory or allergic disease and had normal spirometric results. The study was approved by the Leicestershire Research Ethics Committee. Informed consent was obtained from all subjects.

### Immunohistochemistry

Mucosal biopsy specimens were processed into glycol methacrylate (Polysciences, Northampton, United Kingdom). Two-micrometer sections were stained with antibodies: DP2/CRTH2 (rabbit polyclonal against sequence CAASPQTGPLNRALSSTSS, 1 μg/mL; AstraZeneca, London, United Kingdom) with staining confirmed by an alternative antibody to DP2/CRTH2 OPA1-15328 (5 μg/mL; Thermo Fisher Scientific, Leicestershire, United Kingdom), mast cell tryptase (IR640; Dako, Cambridge, United Kingdom), CD3 (M7254, 5 μg/mL; Dako), major basic protein (MON6008, 1.3 μg/mL; Monosan, Newmarket, United Kingdom), neutrophil elastase (M0752, 0.02 μg/mL; Dako), CD4 (M7310, 5 μg/mL), CD8 (M7103, 1 μg/mL), MUC5AC (Ab24070, 1 μg/mL; Abcam, Cambridge, United Kingdom), pancytokeratin (M0821, 1 μg/mL; Dako), involucrin (Ab68, 0.75 μg/mL; Abcam), or isotype controls (Dako). The EnVision FLEX kit (Dako) was used. Colocalization was undertaken with sequential sections, as described previously.^21^ Positively stained nucleated cells were enumerated per square millimeter of submucosal area, per 10 mm^2^ of total epithelial area, or per millimeter of ALI culture length by a blinded observer. Grading criteria were derived for histology of biopsy specimens and area of involucrin-positive staining. Grading was carried out on 2 separate occasions by a blinded observer.

### Cell culture

Epithelial cells were derived from bronchial brushings of asthmatic patients. Healthy control cells were derived either from bronchial brushings of healthy control subjects or bought from Epithelix (Genève, Switzerland). Fully differentiated epithelial cells were purchased as MucilAir-ALI cultures and grown in bronchial epithelial media (Epithelix).The following treatments (final concentrations) were added to the basal media of duplicate cultures for 24, 48, or 72 hours: DP2 agonist; 13, 14-dihydro-15-keto prostaglandin D_2_ (DK-PGD_2_; 100 nmol/L; Cayman chemicals, Cambridge, United Kingdom); dimethyl sulfoxide (vehicle control; 1 μmol/L; Sigma, St Louis, Mo); or AZD6430 (1 μmol/L; AstraZeneca; concentration 500-fold above its Ki for human DP2). AZD6430 has excellent selectivity. It was tested in more than 100 assays, and the only significant, although very low-affinity, activities were observed at the following receptors and enzymes: D prostanoid receptor 1 (DP1; pIC_50_ = 5.5), thromboxane receptor (pIC_50_ = 5.4), angiotensin type 2 receptor (pIC_50_ = 5.4), aldose reductase (pIC_50_ = 5.2), and COX1/2 (pIC_50_ = 5.5). AZD6430 thus showed at least 1500-fold selectivity over all other targets tested: IL-13, 100 ng/mL (R&D Systems, Abingdon, United Kingdom); TGF-β1, 10 ng/mL (Miltenyi Biotec, Bergisch Gladbach, Germany).

### Epithelial cell expression

Extracellular and intracellular (0.1% saponin) DP2 expression was assessed with DP2-PE antibody relative to isotype control (Rat-PE isotype) with the FACSAria (BD Biosciences, Oxford, United Kingdom). The effects of corticosteroids on DP2 expression were investigated, as described above, by incubating healthy control cells with 1 μmol/L fluticasone propionate (Sigma) for 24 hours. The effects of 100 nmol/L DK-PGD_2_ for 24 hours on DP2 expression were also assessed. For the study of DP2 expression in submerged cells grown in chamber slides, cells were fixed, stained with AstraZeneca DP2 antibody, and detected with anti-rabbit Alexa Fluor 488 (Invitrogen, Paisley, United Kingdom).

### Quantitative RT-PCR

The RNAqueous-4PCR kit (Ambion, Life Technologies, Grand Island, NY) was used for RNA preparation, and the RETROScript cDNA synthesis kit (Ambion) was used for cDNA preparation. TaqMan reagents used DP2 (Hs01867513), DP1 (Hs00235003), MUC5AC (Hs00873651), and 18S (Hs03928985; Applied Biosystems, Warrington, United Kingdom). The Stratagene Mx3000P (Stratagene, La Jolla, Calif) was used. Data were generated with the standard curve method normalized to the 18S housekeeping gene.

### Cell migration

The Oris Cell Migration Kit was used (tebu-bio, Peterborough, United Kingdom). Triplicate repeats for vehicle control (1 μmol/L dimethyl sulfoxide), 100 nmol/L DK-PGD_2_ or 100 nmol/L DK-PGD_2_, and 1 μmol/L AZD6430 were added for 24 hours in 5 healthy control donors and 5 asthmatic donors. The concentrations of 500 nmol/L and 1 μmol/L DK-PGD_2_ were tested in cells from 5 healthy control donors. TGF-β1 (10 ng/mL) and fibroblast growth factor (25 ng/mL; R&D Systems) were used as positive controls in cells from 5 healthy control donors and 2 asthmatic donors. Cells were fixed and labeled with Hoechst nuclear dye (Invitrogen, Carlsbad, Calif). The number of cells migrated into the migration zone was counted by a blinded observer.

### Calcium assay

Calcium responses to 1 μmol/L DK-PGD_2_, 1 μmol/L DK-PGD_2_ and 1 μmol/L AZD6430, and 1 μmol/L ionomycin (Sigma) were assessed in Fura-2 (Invitrogen)–loaded cells, as described previously.^22^

### Analysis

Statistical analysis was performed with PRISM software, version 6 (GraphPad Software, La Jolla, Calif). Parametric data were analyzed with 1- or 2-way ANOVA, Tukey posttest correction for intergroup comparison, or the paired *t* test. Nonparametric data were analyzed with the Kruskal-Wallis test and the Dunn test for *post hoc* comparison. The Spearman correlation test was used for correlation analysis. A *P* value of less than .05 was considered significant.

## Results

### Immunohistochemistry staining for DP2 on biopsy specimens

**Fig E1:**
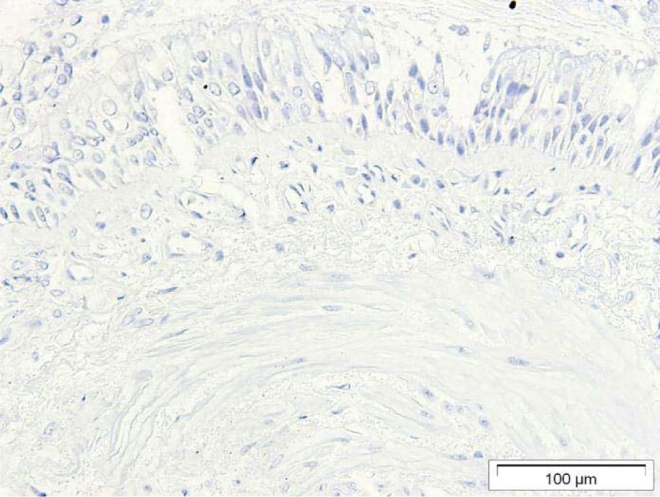
Images are shown at ×200 magnification. DP2 expression in the presence of corresponding blocking peptide is shown. Note the lack of staining.

**Fig E2:**
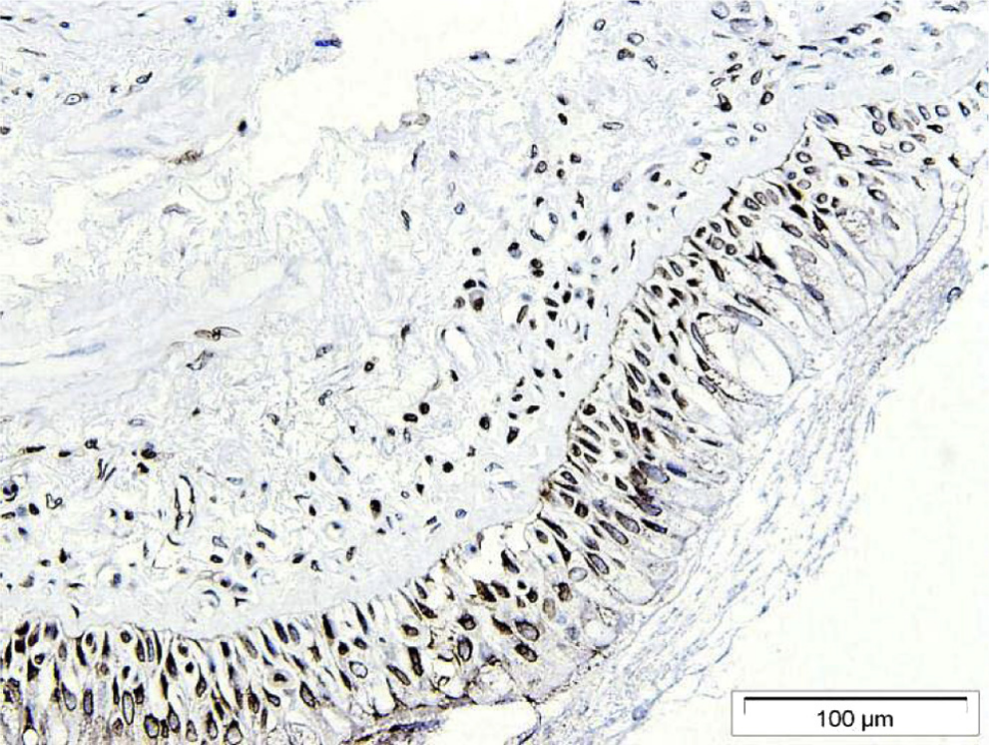
Images are shown at ×200 magnification. DP2 expression (brown staining) on a biopsy specimen from a patient with mild asthma with the AstraZeneca-sourced antibody is shown.

**Fig E3:**
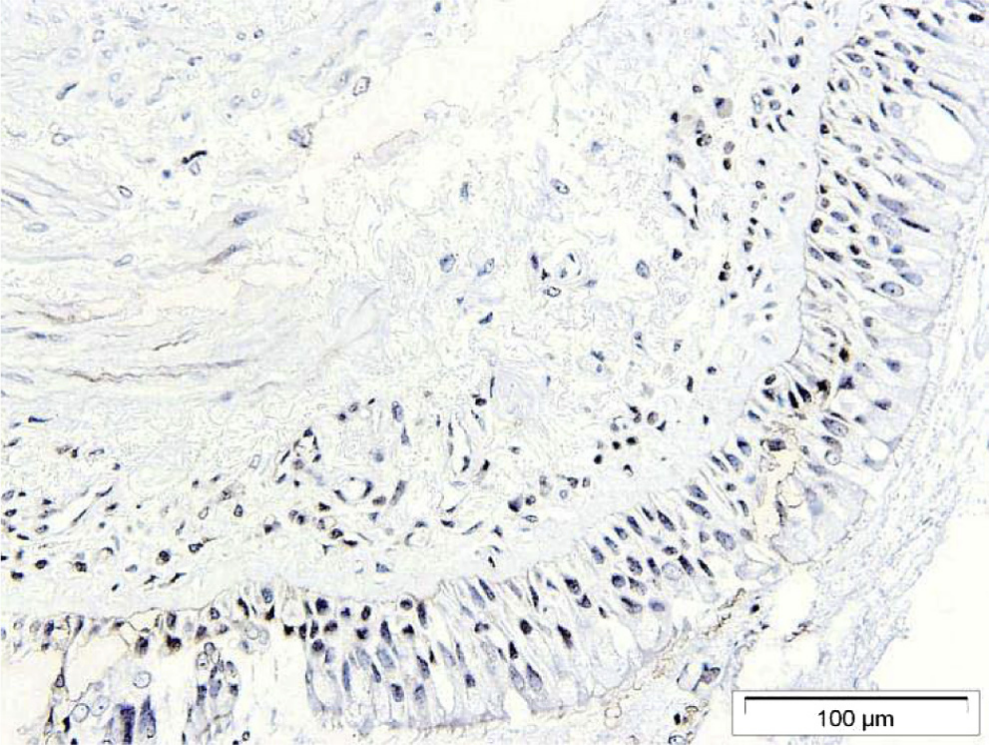
Images are shown at ×200 magnification. DP2 expression (brown staining) on the same biopsy specimen as Fig E2 with the Thermo Fisher Scientific OPA1-15328 antibody is shown.

**Fig 1:**
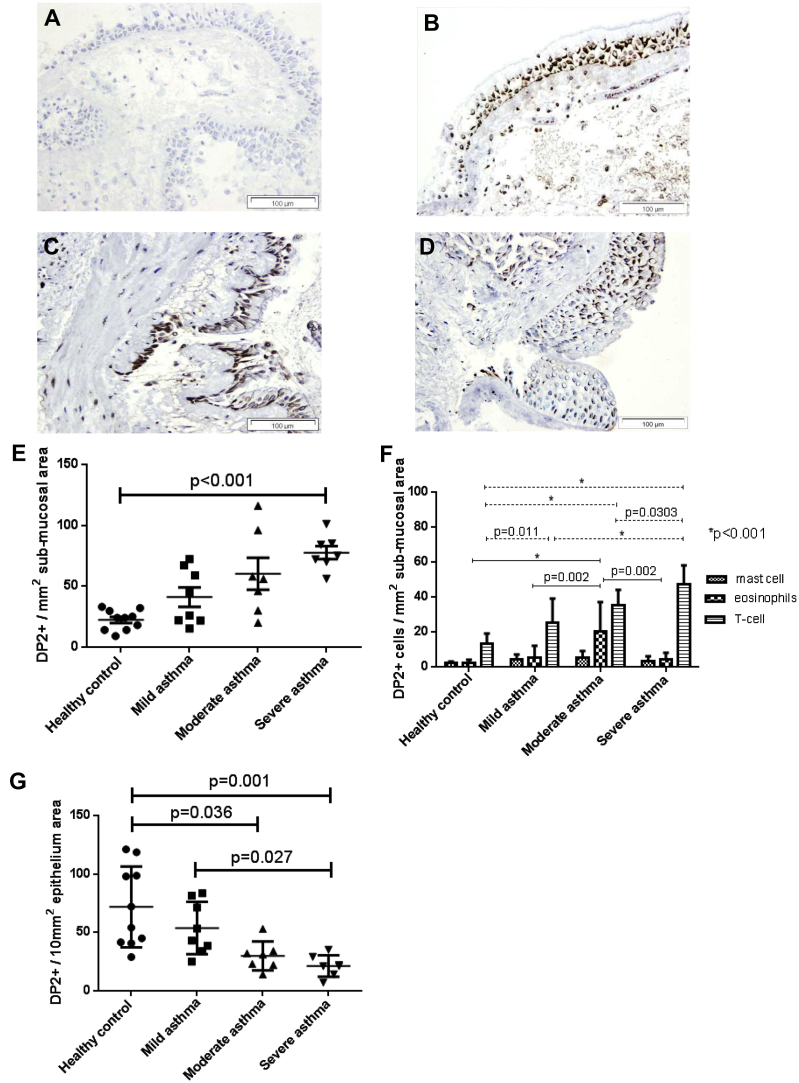
Images are shown at ×200 magnification. **A-D,** Examples of DP2 expression (brown staining) on epithelial cells and inflammatory cells within the bronchial submucosa in rabbit immunoglobulin fraction negative control demonstrating a lack of any positive staining (Fig 1, *A*), a healthy control subject (Fig 1, *B*), a patient with mild asthma (Fig 1, *C*), and a patient with severe asthma (Fig 1, *D*). **E,** Dot plot of DP2^+^ inflammatory cells within the submucosa of healthy control subjects and patients with mild, moderate, and severe asthma. *P* values are based on the Kruskal-Wallis test. Overall *P* < .001. *P* values shown in the figure are based on the Dunn *post hoc* test. **F,** Numbers of DP2^+^ mast cells (mast cell tryptase positive), eosinophils (major basic protein positive), and T cells (CD3^+^), as assessed by means of colocalization of sequential sections. *P* values are based on 2-way ANOVA. Overall *P* < .001. *P* values shown in the figure based on the Tukey *post hoc* test. **G,** Dot plot of DP2^+^ epithelial cells in healthy control subjects and patients with moderate and severe asthma. *P* values are based on the Kruskal-Wallis test. Overall *P* < .001. *P* values shown in the figure are based on the Dunn *post hoc* test.

Clinical characteristics of the patients with mild, moderate, or severe asthma and healthy control subjects are shown in Table I. Groups were well matched for age and smoking history. Asthmatic patients had impaired lung function and evidence of eosinophilic airway inflammation. Representative examples of DP2 expression in bronchial biopsy specimens from asthmatic patients and healthy control subjects are shown (Fig 1, *A-D*). No staining was seen for the isotype control (Fig 1, *A*) or when the antibody was incubated with a blocking peptide (see Fig E1 in this article's Online Repository at www.jacionline.org). Expression with a commercially available DP2 antibody was similar (see Figs E2 and E3 in this article's Online Repository at www.jacionline.org).

DP2 expression was observed on inflammatory cells within the submucosa for biopsy specimens from asthmatic patients and those from healthy control subjects. Quantification of DP2^+^ cells within the submucosa demonstrated a significant increase in biopsy specimens from patients with severe asthma compared with that seen in biopsy specimens from healthy control subjects (mean [SEM]: 78 [5] vs 22 [3] cells/mm^2^ submucosa, *P* < .001; Fig 1, *E*). By using serial section staining, DP2 was found to colocalize with a subset of CD3^+^ T cells, major basic protein–positive eosinophils, and tryptase-positive mast cells (Fig 1, *F*). DP2^+^ submucosal cells were most commonly T cells. The number of DP2^+^CD3^+^ cells was significantly increased in biopsy specimens from patients with mild, moderate, and severe asthma compared with that seen in specimens from healthy control subjects (mean [SEM]: 25 [5] vs 14 [2] mm^2^ submucosa, *P* = .011; 33 [3] vs 14 [2] mm^2^ submucosa, *P* < .001; and 47 [4] vs 14 [2] mm^2^ submucosa, respectively; *P* < .001) and in biopsy specimens from patients with severe asthma compared with those from patients with mild and moderate asthma (mean [SEM]: 47 [4] vs 25 [5] mm^2^ submucosa, *P* < .001; 47 [4] vs 33 [3] mm^2^ submucosa, *P* = .030). In a subset of asthmatic patients (n = 12) and healthy control subjects (n = 5), the DP2^+^CD3^+^ phenotype was investigated further by using CD4 and CD8 markers. The number of DP2^+^CD4^+^ cells was significantly increased in asthmatic patients compared with that seen in healthy control subjects (mean [SEM]: 15 [3] vs 4 [1] mm^2^ submucosa, *P* = .002). DP2 expression was also observed for CD8^+^ cells, but only a small proportion of cells and no significant differences between biopsy specimens from healthy control subjects and asthmatic patients were found (data not shown). There was no colocalization of DP2^+^ cells with neutrophils. Positive expression was seen for biopsy specimens from asthmatic patients and healthy control subjects on epithelial cells. The number of DP2^+^ epithelial cells was significantly reduced in biopsy specimens from patients with moderate and severe asthma compared with those from healthy control subjects (Fig 1, *G*; mean [SEM]: 30 [5] vs 72 [11]/10 mm^2^ epithelium, *P* = .036; 21 [3] vs 72 [11]/10 mm^2^ epithelium, *P* = .001) and in biopsy specimens from patients with severe asthma compared with those from patients with mild asthma (mean [SEM]: 21 [3] vs 54 [8]/10 mm^2^ epithelium, *P* = .027). The number of DP2^+^ inflammatory cells and DP2^+^ epithelial cells had reciprocal correlations with total sputum cell counts (*r* = 0.54, *P* = .003; *r* = −0.42, *P* = .028), FEV_1_ percentage bronchodilator response (*r* = 0.36, *P* = .048; *r* = −0.43, *P* = .002), and airway hyperresponsiveness (*r* = −0.586, *P* = .004; *r* = 0.55, *P* = .009), respectively, but not with FEV_1_ percent predicted, FEV_1_/forced vital capacity ratio, or sputum differential cell counts.

**Fig E4:**
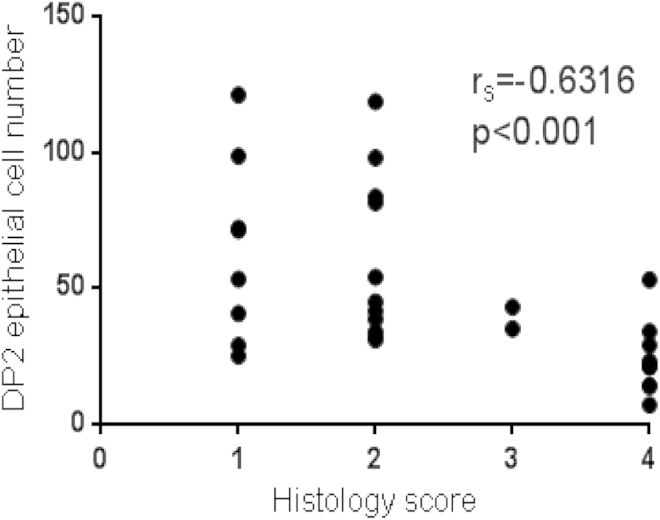
Spearman correlation graph for DP2^+^ epithelial cell numbers versus histology scores.

**Fig E5:**
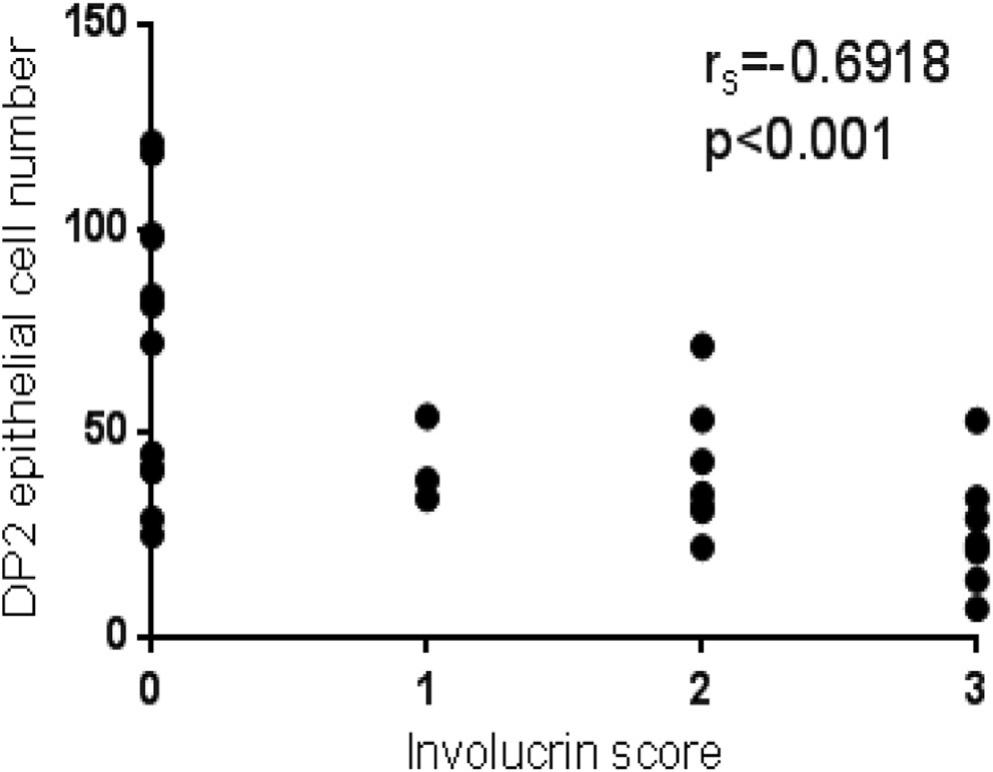
Spearman correlation graph for DP2^+^ epithelial cell numbers versus involucrin scores.

**Fig 2:**
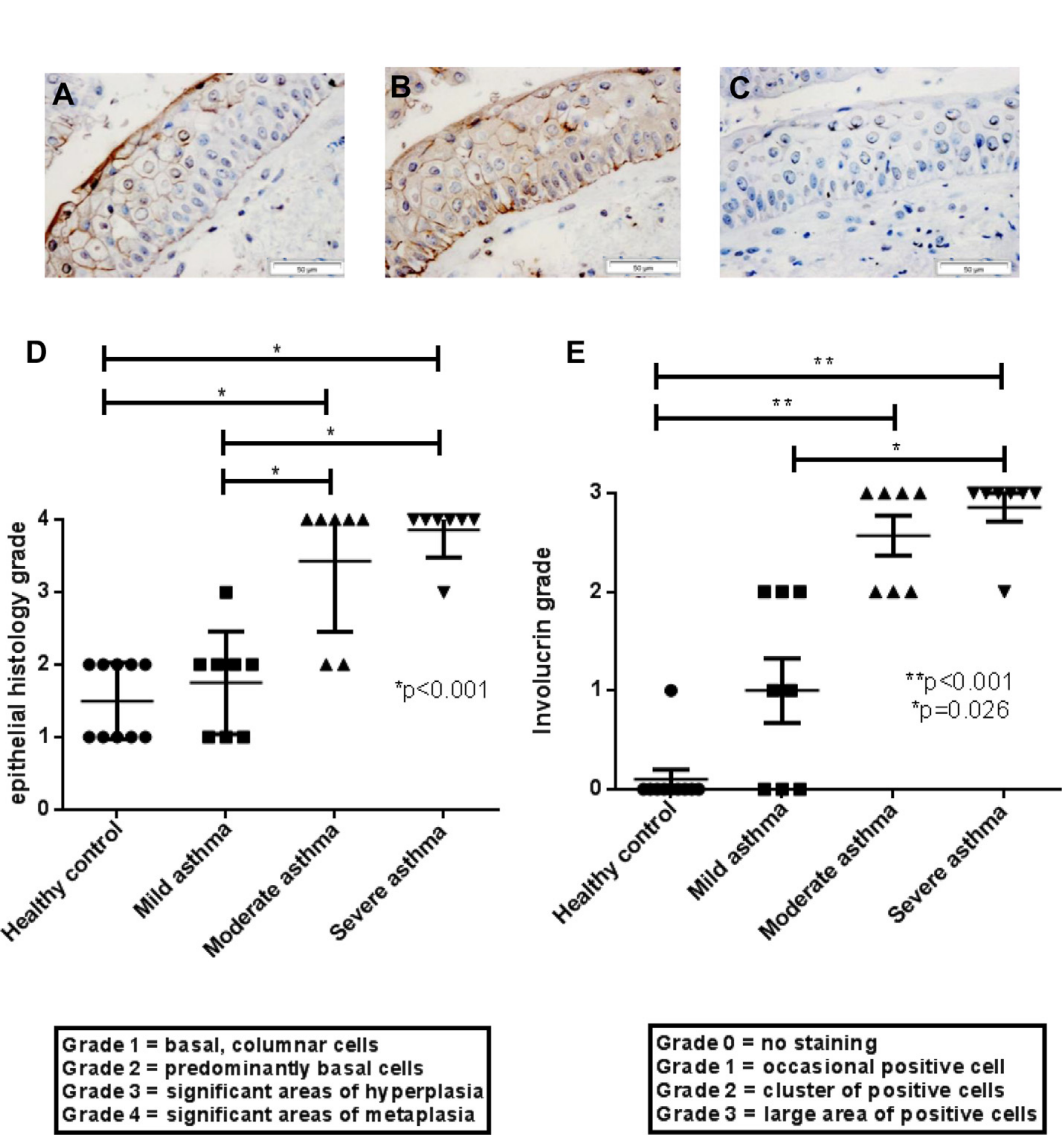
**A-C,** Images (×400 magnification) of serial sections of a severe asthma biopsy specimen after involucrin staining (brown; Fig 2, *A*), pancytokeratin (Fig 2, *B*), and DP2 (Fig 2, *C*). **D,** Epithelial histology grades for biopsy specimens from healthy control subjects and patients with mild, moderate, and severe asthma. *P* values are based on 1-way ANOVA. Overall *P* < .001. *P* values shown in the figure based on the Tukey *post hoc* test. **E,** Grading of involucrin staining for biopsy specimens from healthy control subjects and patients with mild, moderate, and severe asthma. *P* values are based on Kruskal-Wallis tests. Overall *P* < .001. *P* values shown in the figure are based on the Dunn *post hoc* test.

To determine whether a change in phenotype had occurred in the DP2^−^ epithelial cells, we costained cells with involucrin, which was previously described as a reliable marker of a squamous metaplastic phenotype by Araya et al^23^ in the lungs of patients with COPD. We found a lack of DP2 staining on epithelial cells in areas expressing pancytokeratin (used to confirm epithelial origin) and involucrin (Fig 2, *A-C*). Many of the DP2^−^ epithelial cells had a flattened squamous morphology (Fig 2, *C*). These findings suggested that the reduction in DP2^+^ epithelial cell counts was due to a metaplastic change in phenotype of the epithelial cells in the groups with moderate and severe asthma. Epithelial histology for all biopsy specimens were graded according to the criteria shown in Fig 2, *D*. Grading criteria were assessed on 2 separate occasions, and intraclass correlation for all data was strong (Cronbach α = .992, *P* < .001). A significant increase in epithelial histology grade was observed for biopsy specimens from patients with moderate and severe asthma compared with that seen in healthy control samples (median [interquartile range]: grade 4 [2-4] vs grade 1.5 [1-2], *P* < .001; grade 4 [3-4] vs grade 1.5 [1-2], *P* < .001) and biopsy specimens from patients with moderate and severe asthma compared with those from patients with mild asthma (grade 4 [2-4] vs grade 2 [1-3], *P* < .001; grade 4 [3-4] vs grade 2 [1-3], *P* < .001; Fig 2, *D*). Quantification of a change in phenotype of some epithelial cells was achieved by using involucrin staining graded with the criteria described in Fig 2, *E*. A significantly higher incidence of involucrin staining was observed for biopsy specimens from patients with moderate and severe asthma compared with healthy control samples (grade 3 [2-3] vs grade 0 [0-1], *P* < .001; grade 3 [2-3] vs grade 0 [0-1], *P* < .001; Fig 2, *E*) and biopsy specimens from patients with severe asthma compared with those from patients with mild asthma (grade 3 [2-3] vs grade 1 [0-2], *P* = .026). The number of DP2^+^ epithelial cells was negatively correlated with both the histology grade and involucrin grade (*r*_*s*_ = −0.63, *r*_*s*_ = −0.69, *P* < .001; see Figs E4 and E5 in this article's Online Repository at www.jacionline.org).

### DP2 expression on cultured epithelial cells

**Fig 3:**
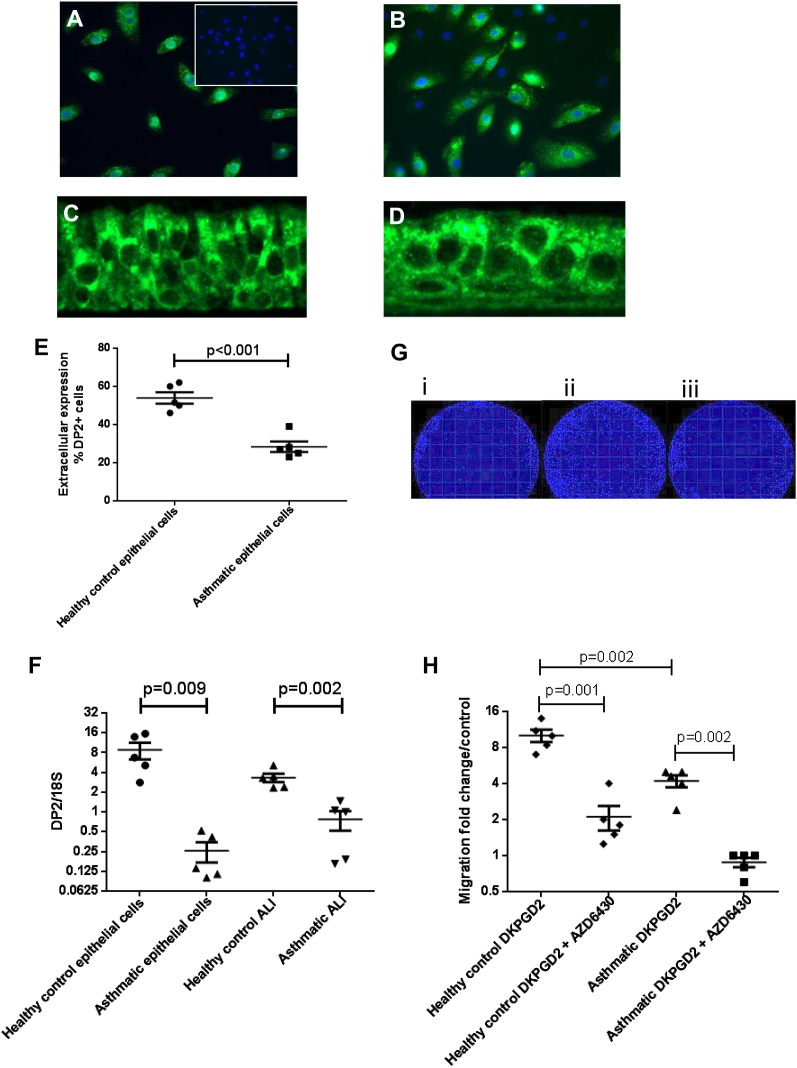
DP2 expression on cultured submerged epithelial cells. Fluorescent cell staining is shown as follows. **A,** Green staining for DP2, with blue 4′-6-diamidino-2-pheynylindole dihydrochloride (DAPI) nuclear staining (cells from healthy control subjects). The *inset* shows a rabbit isotype control with lack of any green staining. **B,** Green staining for DP2, with blue DAPI nuclear staining (cells from asthmatic patients). Note cells with absence of DP2^+^ cells (green) staining. **C,** Green staining for DP2 on ALI culture from healthy control subjects. **D,** Green staining for DP2 on ALI culture from asthmatic patients. **E,** Percentage of DP2^+^ epithelial cells of extracellular expression assessed by means of fluorescence-activated cell sorting. *P* values are based on unpaired 2-tailed *t* tests. **F,** DP2 mRNA expression normalized to the 18S housekeeping gene for epithelial cells. *P* values are based on unpaired 2-tailed *t* tests. **G,***i*, Hoechst-positive epithelial cells within the migration zone for vehicle control. *ii*, Hoechst-positive epithelial cells within the migration zone for 100 nmol/L DK-PGD_2_ treatment. *iii*, Hoechst-positive epithelial cells within the migration zone for 100 nmol/L DK-PGD_2_ plus AZD6430 treatment. **H,** Dot plot of cell migration fold change over vehicle control for both cells from healthy control subjects and those from asthmatic patients with 100 nmol/L DK-PGD_2_ and 100 nmol/L DK-PGD_2_ plus 1 μmol/L AZD6430. *P* values are based on paired 2-tailed *t* tests.

To investigate whether differences in DP2 expression *in vivo* also existed *in vitro*, we characterized the expression of DP2 on cultured epithelial cells taken from healthy subject and asthmatic patients. All asthmatic donors used had moderate-to-severe disease (Global Initiative for Asthma treatment steps 3-5). Fluorescent DP2 cell staining was associated with submerged epithelial cells from healthy subjects and asthmatic patients (Fig 3, *A* and *B*). For epithelial cells from asthmatic patients, there were some cells that were DP2^−^ (4′-6-diamidino-2-pheynylindole dihydrochloride–positive but DP2^−^; Fig 3, *B*). No DP2 staining was observed with the isotype control (Fig 3, *A*, insert). DP2 expression was present on epithelial cells grown in an ALI format from both healthy (Fig 3, *C*) and asthmatic (Fig 3, *D*) donors.

Extracellular expression analysis of DP2 on submerged epithelial cells showed a significant reduction in the percentage of DP2^+^ cells for the cells from asthmatic patients (mean [SEM]: 28% [6%]) compared with those from healthy control subjects (mean [SEM]: 54% [7%], *P* < .001; Fig 3, *E*). A similar trend was observed for intracellular DP2 expression (mean [SEM]: 60% [4%] for healthy control subjects vs 31% [7%] for asthmatic patients, *P* < .001). For both cells from healthy control subjects and those from asthmatic patients, no significant differences were found between the extracellular and intracellular compartments in the number of DP2^+^ cells (mean [SEM]: 58% [6%] for healthy control subjects vs 60% [4%], *P* = .182; 31% [8%] for asthmatic patients vs 31% [7%], *P* > .999). DK-PGD_2_ did not induce any significant change in intracellular percentage of DP2^+^ cells or expression levels (2-fold [0- to 5-fold] increase in DP2^+^ cell percentage, *P* = .550; 1.6-fold [0.6- to 4-fold] increase in expression, *P* = .185). Similarly, fluticasone propionate had no significant effect on extracellular or intracellular percentages of DP2^+^ cells or levels of expression (extracellular 7.8-fold [4- to 16-fold] increase in DP2^+^ cell percentages, *P* = .157; 1.2-fold [0.7- to 2-fold] increase in expression, *P* = .208; intracellular 2.7-fold [0- to 5-fold] increase in DP2^+^ cell percentages, *P* = .370; 1-fold [0.8- to 1.4 fold] increase in expression, *P* = .423). DP2 mRNA expression in epithelial cells was detected for both cells from healthy subjects and those from asthmatic patients grown in a submerged or ALI culture format, with significantly more DP2 expression associated with healthy epithelial cells (mean [SEM]: 9 [3] vs 0.3 [0.1], *P* = .009; 3 [1] vs 0.8 [0.3], *P* = .002; Fig 3, *F*). Because we did not detect any DP1 mRNA expression on epithelial cells grown in submerged or ALI culture, the role of DP1 was not investigated (data not shown).

### DP2 activation causes migration and calcium responses in epithelial cells

**Fig E6:**
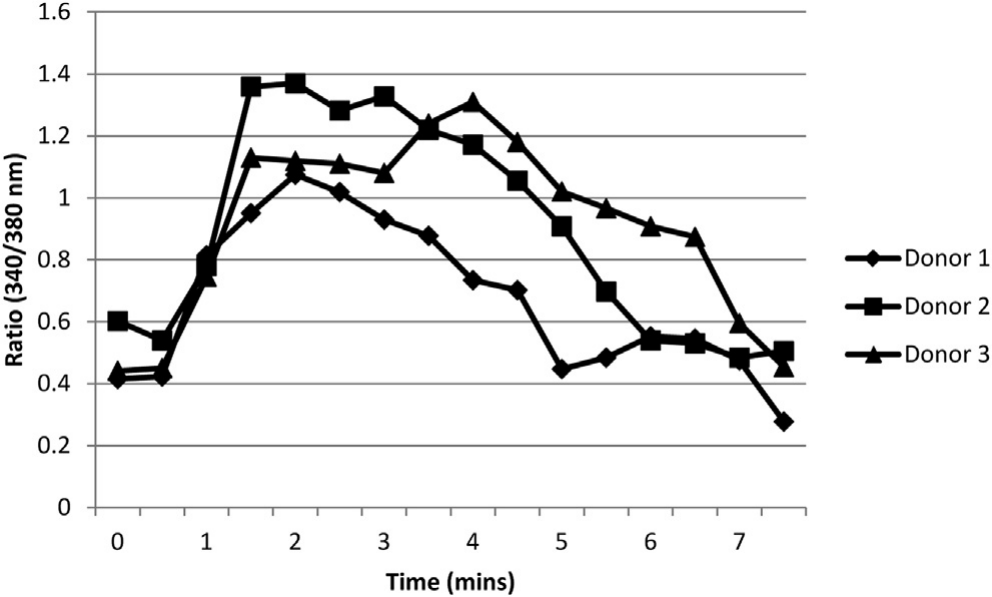
Calcium response curves (Fura-2 ratio, 340/380 nm) for 3 healthy control donors to 1 μmol/L DK-PGD_2_ on submerged bronchial epithelial cells.

**Fig E7:**
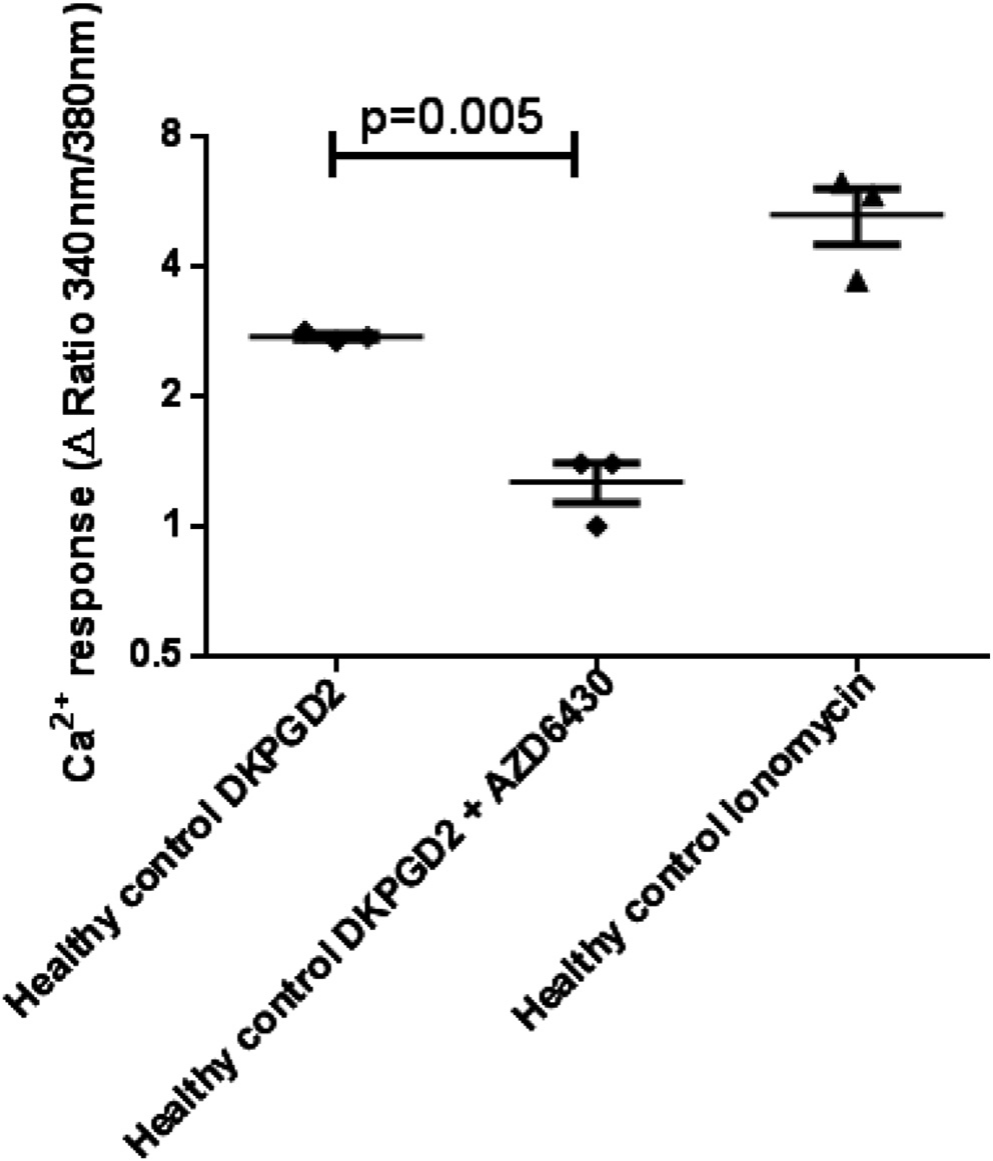
Dot plot to show change in Fura-2 ratio (340/380 nm [peak/basal]) for 3 healthy control donors to 1 μmol/L DK-PGD_2_, 1 μmol/L DK-PGD_2_ plus 1 μmol/L AZD6430, and 1 μmol/L ionomycin on submerged bronchial epithelial cells.

The functional response of DP2 on epithelial cells was assessed by using a cell migration assay. Vehicle treatment caused a small increase in cell migration compared with untreated values, and therefore fold changes were assessed relative to values seen in vehicle-treated cells. DK-PGD_2_ promoted migration of epithelial cells from healthy control subjects (10-fold [7- to 14-fold]) and asthmatic patients (4-fold [3- to 6-fold]), but this was greater in the healthy control subjects than in asthmatic patients (*P* = .002; Fig 3, *G* and *H*). Migration was blocked by the DP2 antagonist AZD6430 in cells from both healthy subjects (10-fold [7- to 14-fold] vs 2-fold [1- to 3-fold], *P* = .001) and asthmatic patients (4-fold [3- to 6-fold] vs 0.8-fold [0.6- to 1-fold], *P* = .002; Fig 3, *G* and *H*). Significant migration was also observed at 500 nmol/L DK-PGD_2_ (10.8-fold [8-14], *P* < .001) and 1 μmol/L DK-PGD_2_ (11-fold [8-15], *P* < .001), but no significant difference was observed between DK-PGD_2_ concentrations. No significant migration was observed in response to the DP2 antagonist AZD6430 (1 μmol/L) alone when compared with vehicle treatment (1.1-fold [0.9-1.4], *P* > .999). A combination of both 10 ng/mL TGF-β1 and 25 ng/mL fibroblast growth factor was used as a positive control for the migration studies, which caused significant migration (7-fold [3- to 12-fold], *P* = .018), a response that was not affected by AZD6430 (5-fold [1.7- to 15-fold], *P* = .102). We also found that 1 μmol/L DK-PGD_2_ elicited calcium responses in Fura-2–loaded epithelial cells, which could be blocked with AZD6430 (see Figs E6 and E7 in this article's Online Repository at www.jacionline.org).

### DP2 activation modulates epithelial differentiation in ALI cultures

**Fig 4:**
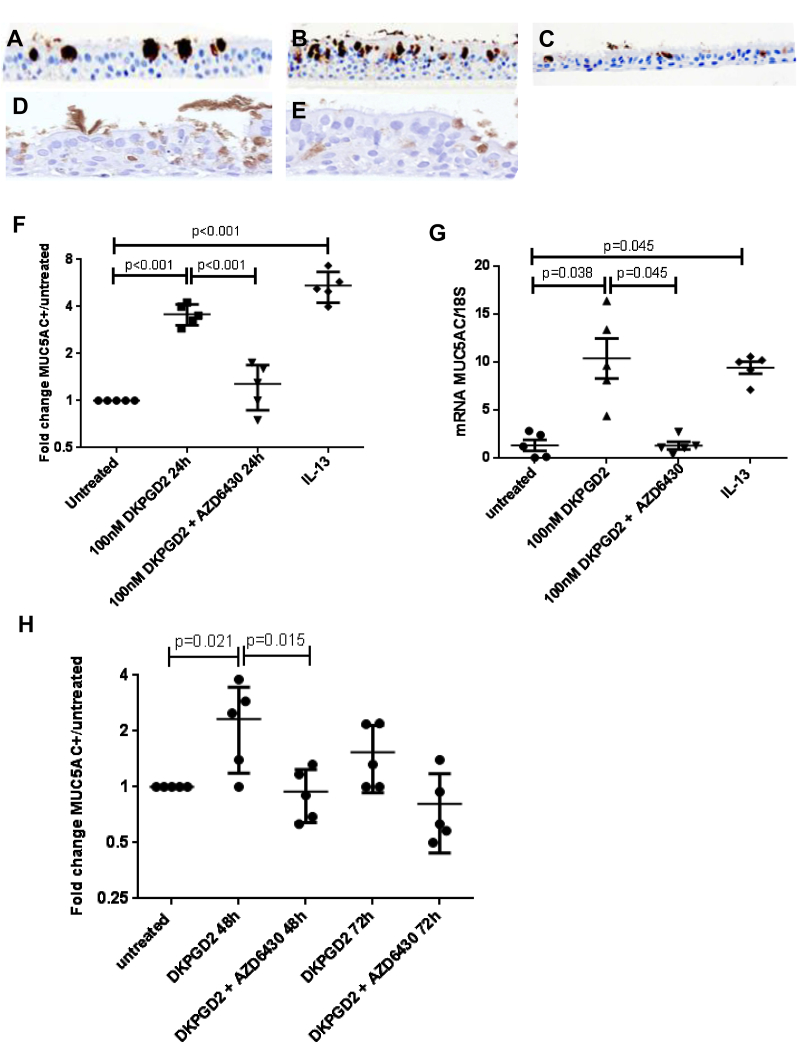
**A-C,** Representative images of healthy control ALI cultures of MUC5AC^+^ staining (brown, ×200 magnification). Results are shown for untreated conditions (Fig 4, *A*) and treatment with 100 nmol/L DK-PGD_2_ for 24 hours (Fig 4, *B*), and 100 nmol/L DK-PGD_2_ plus 1 μmol/L AZD6430 for 24 hours (Fig 4, *C*). **D,** IL-13 (100 ng/mL) for 24 hours. **E,** IL-3 (100 ng/mL) plus AZD6430 (1 μmol/L) for 24 hours. **F,** Dot plot to show fold change in MUC5AC^+^ cells per millimeter of culture over untreated for cultures with 24-hour treatment. *P* values are based on 1-way ANOVA. Overall *P* < .001. *P* values shown in the figure are based on the Tukey *post hoc* test. **G,** Dot plot to show mRNA expression for MUC5AC normalized to 18S expression for cultures with 24-hour treatment. *P* values are based on Kruskal-Wallis tests. Overall *P* = .003. *P* values shown in the figure are based on the Dunn *post hoc* test. **H,** Dot plot to show quantitation of MUC5AC staining for cultures with 48 and 72 hours of treatment. *P* values are based on 1-way ANOVA. Overall *P* = .005. *P* values shown in the figure are based on the Tukey *post hoc* test.

The linkage of DP2 expression with the epithelial phenotype within biopsy specimens led us to hypothesize that DP2 activation might play a role in epithelial differentiation. To investigate this, ALI cultures were used because they contain cells in variable states of differentiation. Five separate donors of healthy ALI cultures were treated with vehicle control, DK-PGD_2_, or DK-PGD_2_ and AZD6430. Treatment for 24 hours with DK-PGD_2_ produced an increase in goblet cell numbers quantified by using MUC5AC^+^ staining, which could be blocked with AZD6430 (Fig 4, *A*, *C*, and *F*). No differences were observed between untreated and vehicle control–treated cultures. A significant fold increase in the number of MUC5AC^+^ cells was observed with DK-PGD_2_ treatment when compared with untreated ALI (4-fold [3- to 4-fold] increase, *P* ≤ .001), which decreased significantly in the presence of AZD6430 (Fig 4, *B*, *C*, and *F*). IL-13, which was used as a positive control, caused a significant fold increase in the number of MUC5AC^+^ cells compared with untreated values (IL-13: 5-fold [4- to 7-fold] increase, *P* < .001; Fig 4, *D* and *F*). AZD6430 did not affect IL-13 responses (5.6-fold [4- to 7-fold] vs 5-fold [4- to 7-fold] with IL-13 alone; Fig 4, *D* and *E*). MUC5AC mRNA analysis showed similar results to the protein expression (Fig 4, *G*). More chronic effects of DK-PGD_2_ incubation were also assessed at 48 and 72 hours. An increase in MUC5AC^+^ cell numbers compared with untreated values was maintained at 48 hours of DK-PGD_2_ (2-fold [1- to 4-fold], *P* = .021), but this effect diminished at 72 hours (Fig 4, *H*).

**Fig 5:**
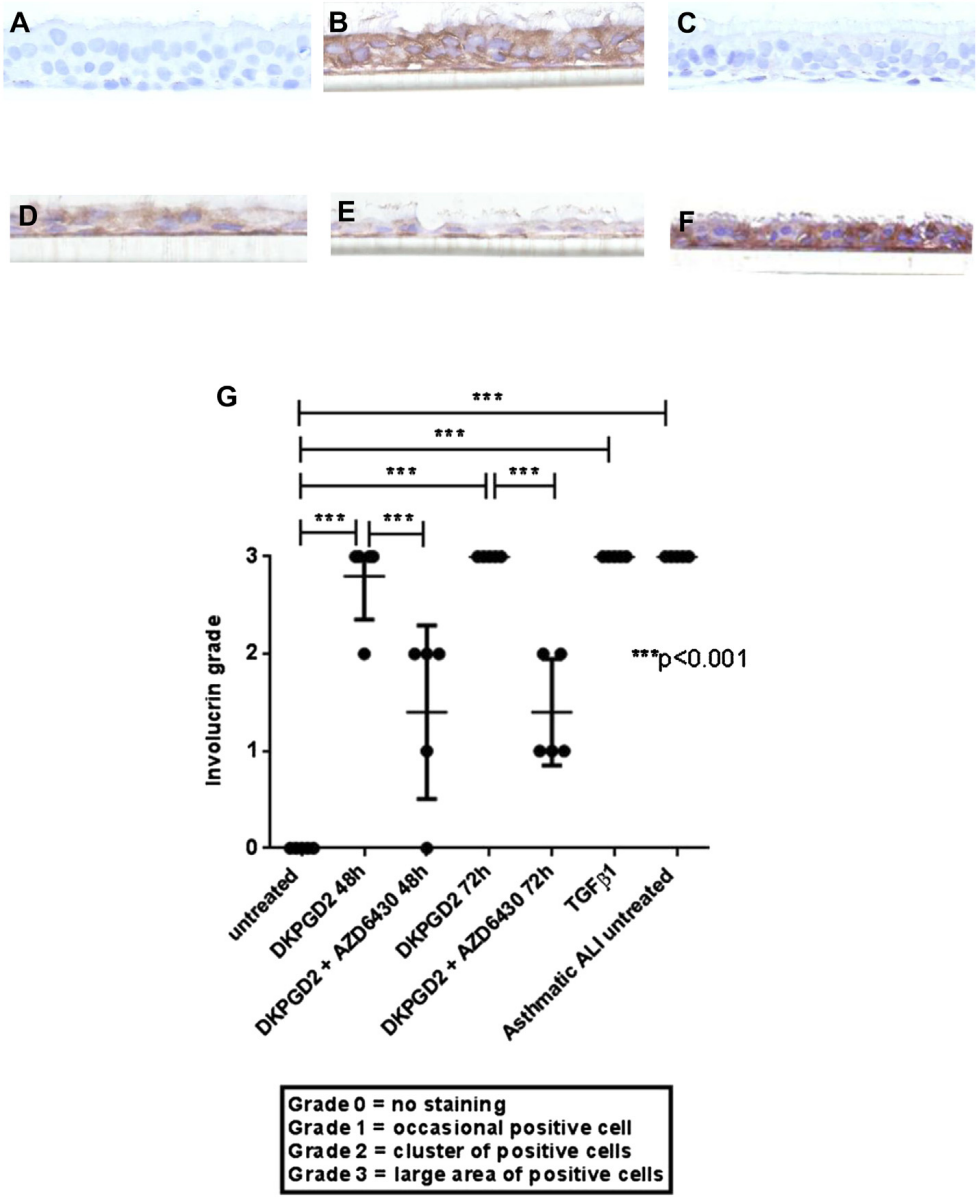
**A-E,** Representative images of ALI cultures from healthy control subjects of involucrin-positive staining (brown, ×400 magnification). Results are shown for untreated conditions (Fig 5, *A*) and treatment with 100 nmol/L DK-PGD_2_ for 48 hours (Fig 5, *B*), and 100 nmol/L DK-PGD_2_ plus 1 μmol/L AZD6430 for 48 hours (Fig 5, *C*). **D,** TGF-β1 (10 ng/mL) for 72 hours. **E,** TGF-β1 (10 ng/mL) plus AZD6430 (1 μmol/L) for 72 hours. **F,** Representative image of involucrin-positive staining for an ALI culture from an untreated asthmatic patient. **G,** Dot blot to show quantitation of involucrin staining for cultures with 48- and 72-hour treatments. *P* values are based on 1-way ANOVA. Overall *P* < .001. *P* values shown in the figure are based on the Tukey *post hoc* test.

Involucrin immunohistochemistry staining was used to further assess the differentiation status of the ALI after more chronic DK-PGD_2_ treatment. Staining was graded according to the same criteria as used for the biopsy specimens. A significant increase in involucrin staining was seen for the ALI cultures treated with DK-PGD_2_ at 48- and 72-hour treatments (untreated: grade 0 [0-0], DK-PGD_2_ 48-hour: grade 3 [2-3], *P* < .001, DK-PGD_2_ 72-hour: grade 3 [3-3]; *P* < .001; Fig 5, *A*, *B*, and *G*). AZD6430 significantly decreased involucrin staining compared with DK-PGD_2_ alone (Fig 5, *B*, *C*, and *G*). TGF-β_1_, which was used as a positive control, caused a significant increase in involucrin staining (untreated: grade 0 [0-0] vs TGF-β_1_: grade 3 [3-3]; *P* < .001; Fig 5, *D* and *G*). The effects of TGF-β1 were unaffected by AZD6430 (TGF-β1: grade 3 [3-3] vs TGF-β1 + AZD6430: grade 3 [3-3]; Fig 5, *D* and *E*). Involucrin staining was also assessed for 5 ALI cultures from asthmatic donors. Cultures from asthmatic donors had significantly higher involucrin grades than the untreated healthy control cultures (untreated: grade 0 [0-0] vs asthmatic: grade 3 [3-3]; *P* < .001; Fig 5, *F* and *G*).

## Discussion

Here we present compelling evidence that DP2 is differentially expressed on inflammatory and epithelial cells in the airways of patients with moderate-to-severe asthma when compared with those of healthy control subjects. More importantly, accumulation of DP2^+^ T cells in the bronchial submucosa was closely associated with asthma severity. We also show that DP2 activation in epithelial cells induces proremodeling responses. These findings demonstrate that activation of DP2 in T cells and the epithelium has the potential to drive key features of severe asthma.

Few studies have investigated the expression of DP2 in the airways of patients with lung diseases and how this expression correlates with disease severity. Previous studies have shown increased numbers of DP2^+^ T cells in the nasal mucosa of allergic compared with nonallergic subjects^14^ and in the bronchoalveolar lavage fluid cells of patients with severe asthma compared with numbers seen in healthy subjects.^6,24^ Our study is the first to demonstrate an increased infiltration of DP2^+^ T cells in the bronchial submucosa of patients with moderate-to-severe asthma when compared with values in healthy subjects. Further analysis of the DP2^+^ T cells on a subset of biopsy specimens demonstrated that the majority of T cells were CD4^+^, although a small proportion of CD8^+^ T cells were also DP2^+^. In addition, we speculate that type 2 innate lymphoid cells contribute to the total number of DP2^+^ T cells because these have been previously been found to express DP2.^25,26^ DP2 has been found to delay apoptosis of T_H_2 lymphocytes,^27^ and the findings from our study support the concept that this action might cause T cells to be retained within the submucosa of the airways. Increased numbers of DP2^+^ eosinophils were found in biopsy specimens from patients with moderate asthma. DP2 antagonists have been found to reduce sputum eosinophil numbers in allergen-challenged steroid-naive asthmatic patients^12^ and nasal eosinophil numbers in patients with allergic rhinitis.^9^ Our data suggest that there is a potential that DP2 antagonists can affect tissue eosinophil numbers in patients with moderate asthma. Although DP2 was also found on mast cells (with a lack of expression on neutrophils), there was no significant difference between healthy subjects and asthmatic patients in DP2 expression on these cell types. The contribution of DP2 in the pathogenesis of asthma has not been completely elucidated. Activation of DP2 on T_H_2 cells has been shown to cause an increase in the ability of these cells to produce IL-2, IL-4, IL-5, and IL-13.^28,29^ In turn, these cytokines could regulate key features of severe asthma, as suggested by preclinical studies showing that DP2 antagonism significantly reduced allergen-induced inflammatory changes within mouse airways.^18^ Therefore activation of accumulated DP2^+^ T cells within the airways of asthmatic patients is likely to play a significant role in the pathogenesis of allergic asthma through proinflammatory actions.

There is a wealth of literature suggesting that epithelial cells contribute to remodeling changes within the airways of asthmatic patients (as reviewed by Davies^30^). Epithelial cells are more stressed in asthmatic patients, showing upregulation of activated transcription factors,^31^ and activated repair processes are evidenced by increased epithelial growth factor receptor^32,33^ and a persistently defective barrier.^34^ We found that DP2 was expressed on epithelial cells within biopsy specimens from asthmatic patients. Although previous publications have described the expression of DP2 on cultured normal human epithelial cells and H292 cells,^35^ our report is the first to demonstrate the *in vivo* expression of DP2 on epithelial cells within bronchial biopsy specimens. A recent report has described expression of DP2 on epithelial cells in lung volume reduction tissue from patients with COPD,^17^ but whether this expression was associated with disease severity was not investigated because no healthy control tissue was included. We have shown that the numbers of DP2^+^ epithelial cells were significantly decreased in the airway epithelium in patients with moderate-to-severe asthma. Further investigations led us to demonstrate that the epithelial cell phenotype in biopsy specimens of patients with moderate-to-severe asthma was dramatically altered when compared with that in healthy control subjects. We found that there were frequent areas of squamous metaplasia in patients with moderate-to-severe asthma when using the involucrin marker previously described in patients with COPD.^23^ Interestingly, quantification of epithelial changes to those seen with a metaplastic phenotype inversely correlated with DP2 expression. Squamous metaplasia and mucous cell metaplasia are the most common metaplastic features associated with epithelial tissue.^36^ More importantly, squamous metaplasia has been found to correlate with the severity of airway obstruction^37^ and to increase with the severity of COPD,^23^ possibly related to cigarette smoke exposure because squamous metaplasia is more frequent in asthmatic patients who smoke.^38^ In this study squamous metaplasia was increased in the moderate-to-severe asthma cohort without a difference in smoking status between the groups. This suggests that there might be other factors independent of cigarette smoke which can contribute to the induction of squamous metaplasia in airway epithelium. Our data indicate that in patients with moderate-to-severe asthma, a phenotype shift of epithelial cells can occur, which influences DP2 expression. Interestingly, differential expression between healthy control subjects and patients with moderate-to-severe asthma was also maintained for epithelial cells when grown in culture. This finding could suggest that cultured epithelial cells from asthmatic patients have an intrinsically altered phenotype, an observation that has been suggested in previous studies using epithelial cells from children with asthma.^39^ In our study DP2 was found to have intracellular and extracellular expression on epithelial cells similar to that seen in previous studies for this receptor.^14,35^ DP2 activation caused functional consequences on epithelial cells that were likely mediated through cell-surface receptors. However, intracellular receptor activation can also occur, as has been reported for other G protein–coupled receptors,^40^ and its functional importance requires further study.

The novel observation of DP2 expression on bronchial epithelial cells directed further investigation into the functional effects of DP2 activation on cultured bronchial epithelial cells. DP2 activation through PGD_2_ has been shown to cause cell migration in T_H_2 cells, basophils, and eosinophils.^8,41^ In the current study we found that DP2 activation with the DP2-selective agonist DK-PGD_2_^42,43^ also caused migration of cells from both asthmatic patients and healthy subjects, an effect that was blocked with a DP2-selective antagonist. However, although the antagonist was highly selective, it had low affinity for other receptors and enzymes, such as thromboxane receptor, and thus we cannot fully exclude off-target effects. Migration was more pronounced in cells from healthy control subjects, possibly because of the difference in cell-surface receptor expression. We recognize that within this study, a dose-response curve was not fully explored in part because of limitations of cell numbers, but increasing concentrations of DK-PGD_2_ up to 1 μmol/L revealed that maximal migratory responses were obtained. Future studies comparing different concentrations of DK-PGD_2_ with more potent DP2 agonists, such as 15(R)-15-methyl-PGD_2_, might help in uncovering differences between the asthmatic and healthy states. The existence of functional DP2 on the epithelium is supported with work in mouse models, where DP2 antagonists have been found to influence mucous cell metaplasia and epithelial cell hyperplasia in response to cigarette smoke^19^ or allergen stimulation.^44^ Using the ALI culture system, which closely mimics the *in vivo* environment,^45^ we provide additional evidence for a role of DP2 in driving phenotype changes of the epithelial cells by showing that DK-PGD_2_ treatment induced not only increased goblet cell numbers when exposed acutely but also increased the area of involucrin expression in the epithelium with more chronic treatment. The positive controls used in this study validated these responses, in which IL-13 significantly upregulated the number of MUC5AC^+^ cells^46^ and TGF-β1 significantly increased the amount of involucrin expression.^47^

Epithelial cells are thought to be highly plastic in that they can rapidly change their phenotype in response to insult.^48^ The classical repair response of epithelial cells to injury is thought to consist of a number of steps. These include transient mucus release, shedding of columnar epithelial cells, spreading and migration of basal epithelial cells, and induction of squamous metaplasia through progressive redifferentiation, ultimately leading to regeneration of the mucociliary epithelium.^48-50^ The expression of DP2 on basal and columnar epithelial cells and the findings that DP2 activation can cause many of these repair step processes could indicate that this receptor plays a key role in the maintenance and repair of the epithelial barrier. In asthmatic patients, in whom there is an increased presence of PGD_2_,^2,3,6^ it is likely that DP2 activation accelerates these functional responses on epithelial cells, causing an aberrant mucosal barrier phenotype. Such changes can aid progression of disease and make patients more susceptible to respiratory tract infection.^51-53^ Therefore a DP2 antagonist might be useful in decreasing DP2 activation on epithelial cells and restoring normal epithelial differential processes.

In conclusion, we have described the differential expression of DP2 on biopsy specimens from healthy control subjects and asthmatic patients. Biopsy specimens from patients with severe asthma were associated with increased DP2^+^ T-cell numbers within the submucosal compartment and reduced DP2^+^ epithelial cell numbers in areas of epithelial metaplasia. Some of the epithelial features seen in patients with severe asthma could be reproduced by activating DP2 on bronchial epithelial cells, causing cell migration and an increase in numbers of goblet cells and cells of a squamous phenotype. The effects of DP2 activation on epithelial cells might influence airway remodeling processes in asthmatic patients, and end points, such as mucus production, should be considered in future clinical DP2 antagonist studies. We conclude that a DP2 antagonist might not just inhibit infiltration of DP2^+^ inflammatory cells into the airways but might also act on epithelial cells and prevent proremodeling action.

**Key messages**

• DP2 is differentially expressed on inflammatory cells and bronchial epithelial cells in biopsy specimens from patients with asthma compared with those from healthy control subjects.
• DP2 activation on bronchial epithelial cells might contribute to airway remodeling in asthmatic patients.

## Figures and Tables

**Table I:** Clinical characteristics for biopsy specimens used for immunohistochemical analysis

	Healthy control subjects (n = 10)	Patients with mild asthma (n = 8)	Patients with moderate asthma (n = 7)	Patients with severe asthma (n = 7)	*P* value
Age (y)	50 (5)	48 (5)	53 (6)	52 (4)	.94
Male sex (female sex)	7 (3)	3 (5)	2 (6)	2 (5)	.19
Atopy (no.)	4	5	4	6	.31
Smoking history (exsmoker/current smoker/never smoker)	2/0/8	2/0/6	1/0/6	2/0/5	.92
ICS dose (μg BDP eq/d)	0	75 (53)	800 (0)	1565 (148)	<.001
LABA use (%)	0	0	100	100	<.001
Oral corticosteroid use (no.)	0	0	0	3	.01
FEV_1_ (% predicted)	99.9 (4.4)	78.6 (6.8)	79.2 (3.7)	82.0 (8.4)	.01
FEV_1_/FVC (%)	78.6 (2.9)	72.5 (3.8)	72.1 (3.7)	68.7 (3.3)	.22
Bronchodilator reversibility (%)∗	0.5 (5.2)	10.9 (24.6)	12.4 (20.3)	8.9 (8.9)	.04
Total cell count (10^6^ cells/g sputum)∗	ND	1.7 (1.1)	5.5 (5.3)	4.2 (6.1)	.06
Sputum eosinophils (%)∗	ND	3.5 (18.7)	0.4 (1.1)	5.2 (30.8)	.30
Sputum neutrophils (%)∗	ND	47.5 (25.5)	57.4 (67.8)	57.9 (54.0)	.60

Data are expressed as means (SEMs). Comparisons across groups were done by means of ANOVA or the Kruskal-Wallis test. *BDP eq*, Beclomethasone dipropionate equivalent; *FVC*, forced vital capacity; *LABA*, long-acting β_2_-agonist; *ND*, not done.

∗ Median (interquartile range).
